# Evaluation Method of Texture of Glutinous Rice Cakes (Niangao) and Its Key Impact Indicators

**DOI:** 10.3390/foods13040621

**Published:** 2024-02-19

**Authors:** Qingyun Lyu, Xing Wang, Yunzhuo Dang, Lijie Zhu, Lei Chen, Xuedong Wang, Wenping Ding

**Affiliations:** 1 School of Food Science and Engineering, Wuhan Polytechnic University, Wuhan 430023, China; wangxingyyds2024@163.com (X.W.); lqingy2001@163.com (Y.D.); lijiezhu325@126.com (L.Z.); chenleiy@whpu.edu.cn (L.C.); xuedongwuhan@163.com (X.W.); whdingwp@163.com (W.D.); 2 Key Laboratory of Grain and Oil Processing, Ministry of Education, Wuhan 430023, China

**Keywords:** glutinous rice, Niangao, texture properties, PCA, HCA, SLR

## Abstract

This study aimed to find a unique method to assess the textural properties of Niangao (glutinous rice cakes), to determine the relationship between the textural properties of rice cakes and the indicators of glutinous rice, and to identify the key indicators that significantly affect the textural properties of Niangao. The study encompassed the analysis of the chemical composition and pasting characteristics of 22 glutinous rice varieties, revealing the substantial impact of variety on lipid content, straight-chain starch content, and pasting performance. Subsequently, the textural features of the resulting Niangao were subjected to principal component analysis (PCA) to derive a mathematical method for evaluating their textural attributes, with the obtained scores employed in hierarchical cluster analysis (HCA) to identify 12 key textural characteristics. Further analysis using stepwise linear regression (SLR) demonstrated that the regression model incorporating final and peak viscosities of the glutinous rice significantly predicted the composite score of the Niangao’s textural properties. This highlights the importance of final and peak viscosities as key indicators for assessing the textural quality of Niangao.

## 1. Introduction

Rice has properties such as low levels of protein, fat, and fiber, the absence of gluten, and a great amount of easily digested carbohydrates. Rice products are a major food source, especially in China, which is the largest rice producer and consumer in the world. Rice products have many unique attributes, such as ease of digestion, bland taste, and hypoallergenic properties [1]. It could be milled into flour and utilized to produce many kinds of foods, including several types of glutinous rice cake. Niangao, prepared from glutinous rice flour, is a kind of steam-cooked Chinese traditional food. It is famous for its pliable, elastic, and sticky texture and it is usually served as a dessert. Due to the different raw materials and processes of rice cakes and differences in people’s eating habits in different places, it is difficult to have a uniform evaluation standard for the taste of quality Niangao.

The sensory quality of Niangao still remains a challenge, despite numerous attempts to explain the mechanism responsible for differences in Niangao’s quality. It was reported that starch is the major component of the system, a predominant role has been assigned to starch retrogradation, which involves the progressive association of gelatinized starch segments into a more ordered structure. The fracture properties of foods are highly correlated with some sensory texture attributes [2]. Hardness and adhesiveness as estimated by a rapid visco analysis (RVA) are indicators of the textural properties of rice cakes [3]. In addition, changes in these parameters are useful to evaluate and compare the textural properties of glutinous rice cakes. A product texture can be evaluated by physicochemical properties measured objectively and by sensory methods. The relationship between consumer preferences and the texture properties of foods is a key part of the science of texture. Texture is defined as the attributes of foods resulting from a combination of physical properties and perceived as the sense of touch, sight, and hearing. Texture profile analysis has been proven to have correlations with RVA measurements for cooked rice [4]. However, the information on the relationship between the physicochemical and sensory properties of foods is limited in spite of the importance of such information in assessing food quality [5].

Instrumental evaluation is objective, convenient, and reproducible, and is widely used in the evaluation of food quality. The most commonly used method for texture characterization in the evaluation of rice products such as rice cakes is the total texture analysis (TPA) method. Malcolm provides a detailed account of the TPA testing process and elucidates the meaning of the TPA curve [6]. However, the hardness, viscosity, cohesion, elasticity, and chewability obtained using the TPA method can hardly reflect the quality of food directly and comprehensively. Therefore, many studies have adopted reasonable data processing methods, such as principal component analysis [7], to simplify multiple quality indicators into one comprehensive indicator [8].

In China, various products can be processed from glutinous rice, such as glutinous dumplings and Niangao. Each product has different requirements for the characteristics of the raw materials. A decrease in protein content in the raw materials can result in increasing cooking solid losses and a decreasing height-to-diameter ratio of cooked glutinous dumplings [9]. When damaged starch in the raw materials is below 5%, the sweet dumpling qualities showed compact structure, weak water mobility, less water loss, slight cracking, and desirable cooking and texture properties [10]. Therefore, this study systematically investigates the relationship between the characteristics of glutinous rice raw materials and the texture and quality of Niangao, aiming to provide theoretical guidance for the development of specialized glutinous rice flour for Niangao production. The objectives of this research were: (1) to find a unique method to evaluate the texture properties of Niangao; (2) to determine relationships between the texture of Niangao and basic chemical composition and RVA of glutinous rice; and (3) to determine the crucial indicators that affect texture properties of Niangao significantly.

## 2. Materials and Methods

### 2.1. Materials

A total of 22 varieties of glutinous rice were collected, as shown in Table 1. The samples were collected from Henan Huangguo Grain Industry Co., Ltd. (Xinyang, Henan, China) and Yunmeng County Zengdian Grain Purchasing and Marketing Co., Ltd. (Xiaogan, Hubei, China) with raw material origins in Anhui, Heilongjiang, Hubei, and Henan provinces, and one type of glutinous rice was processed from glutinous rice imported from Vietnam. All these materials were packed in a vacuum and stored at 20.0 ± 5.0 °C.

### 2.2. Basic Chemical Composition

Water, protein, lipid, total starch, and amylose content were measured according to a reported method [11]. The water content of glutinous rice was measured by gravimetry at 105 °C until a constant weight was reached. Protein content was determined using the Kjeldahl method with a conversion factor of 5.95 to estimate the protein content. Lipid was extracted without previous drying in Soxhlet with petroleum ether for 6 h. The content of amylose was determined using an Amylose/Amylopectin Kit (K-AMYL 12/16, Megazyme International Ltd., Wicklow, Ireland). The content of the total starch in the different glutinous rice was determined using a Total Starch Assay Kit (K-TSTA-50A/K-TSTA-100A 06/17, Megazyme International Ltd., Wicklow, Ireland). All other chemicals used were of analytical grade, and distilled water was used in the experiment.

### 2.3. Pasting Properties

Weigh 2.5 g of glutinous rice flour (completely dry), add 25 mL of distilled water with a pipette, and mix in an RVA container. The pasting properties were measured by a Rapid Visco Analyzer (RVA Super 4, Newport Scientific Pty Ltd., Warriewood, NSW, Australia) according to a reported method [12].

The suspension was stirred with matching propellers at the standard program. The detailed program condition was as follows: the suspension was held at 50 °C for 1 min and heated to 95 °C over a period of 3.5 min and held at 95 °C for 3 min. The paste was cooled down to 50 °C over another 3.5 min and held at 50 °C for 2 min. Each sample was tested in triplicate.

### 2.4. Preparation of Niangao

The glutinous rice was ground into flour using a flour grinder (HR2200, Yongkang Harui Industry & Trade Co., Ltd., Jinhua, Zhejiang, China). The rice flour was sieved through a 60-mesh screen. The ingredients (100 g of glutinous rice flour, 60 mL of water) were mixed until the dough just combined. Then, the raw Niangao was shaped by a glutinous rice cake machine (XZ-5000A, Guangzhou Xuzhong Food Machinery Co., Ltd., Guangzhou, Guangdong, China) and steamed for 10 min in an electric cooker (CFXB100 Hefei Rongshida Small Household Appliances Co., Ltd., Hefei, Anhui, China) preheated to 100 °C. The Niangao samples were cooled at 20.0 ± 5.0 °C and packaged in polyethylene bags (food packaging usage, purchased from supermarket).

### 2.5. Texture Measurements

Texture measurements were performed using a Texture Analyser (TA-XT2i, Stable Micro System, Ltd., Godalming, UK) with a maximum force of 5 kg to imitate the chewing action of the teeth [13]. Niangao samples, having a thickness of approximately 1.5 cm and 50 cm^2^ area, were prepared using a stainless steel mold and then placed on the platform. A cylinder-type stainless steel probe with a diameter of 45 mm was used to compress each sample to 80% of its original height with a pretest speed of 1.0 mm/s, test speed of 1.0 mm/s, and post-test speed of 2.0 mm/s. On each Niangao, the measurements were carried out on the center of the sample and all analyses were conducted 5 times. Parameters of TPA, namely hardness, adhesiveness, springiness, cohesiveness, gumminess, chewiness, and resilience were calculated using the software provided with the instrument.

### 2.6. Statistical Analysis

Factor analysis of texture properties of 22 kinds of Niangao was used in this paper. PCA was used as an extraction method. The principal component (PC) was extracted if its eigenvalue was beyond 1 [14].

According to the result of HCA, SLRs were performed to obtain the function relationship between the comprehensive scores and indicators of materials. It should be announced that all the values of indicators were required to be standardized in advance.

All samples were analyzed in triplicate. Significance analysis, PCA, cluster analysis, and stepwise regression analysis were conducted using IBM SPSS statistical software 19. Specifically, significance analyses were performed using one-way ANOVA, the significance level was determined by Duncan’s multiple comparative analysis, and the difference was determined as significant when *p* < 0.05.

## 3. Results and Discussion

### 3.1. Basic Chemical Composition and Pasting Properties

Research has demonstrated that the chemical composition of rice cakes significantly affects their characteristics. Additionally, the pasting characteristics were measured to further understand the impact of different types of glutinous rice on the textural properties of rice cakes. From Table 1, it can be seen that the basic chemical composition differed among the different varieties of glutinous rice. Table 2 shows the pasting properties of glutinous rice raw material. The descriptive statistics of chemical compositions and pasting properties of the raw materials are listed in Table 3.

Overall, according to the significance analysis, there were significant differences (*p* < 0.05) observed in the content of water, protein, lipid, total starch, and amylose among different glutinous rice varieties. The total starch content of the 22 glutinous rice varieties ranged from about 78 to 88 percent (*w*/*w*), the coefficient of variation (CV) of the total starch content of glutinous rice samples was the smallest, which was only 2.98%, the water content and the protein content ranged from about 12 to 16 percent(*w*/*w*) and from 7 to 9 percent (*w*/*w*). the CV of water and protein content was 9.13% and 9.18%, respectively, indicating that there was little difference in water content, protein content, and total starch content among different cultivars of glutinous rice; the lipid content and the content of straight-chain starch ranged from about 1 percent (*w*/*w*) and from 1 to 4 percent (*w*/*w*), the CV of lipid and amylose content was 21.59% and 21.26%, respectively, which were higher in the chemical compositions of glutinous rice.

The variation in the basic chemical composition of glutinous rice has a certain influence on the pasting properties and retrogradation properties of glutinous rice flour, thereby affecting the textural quality of Niangao. Research has shown that the protein content in glutinous rice flour significantly affects the appearance quality and cooking characteristics of glutinous dumplings. As the protein and lipid content increase, the height-to-diameter ratios of cooked glutinous dumplings also increase. However, an increase in protein content leads to a decrease in overall acceptability [9]. Therefore, a suitable basic chemical composition of raw materials is necessary for the processing of Niangao.

The CV of pasting temperature was the smallest. The CVs of other pasting properties were all above 30%. According to the significance analysis, there were significant differences (*p* < 0.05) observed in the pasting properties, such as peak viscosity and pasting temperature, among different glutinous rice varieties. The RVA pasting properties are good indicators of rice noodle quality and could be used as indirect measures of rice noodle texture in the early stages of rice breeding programs [15]. In general, a low pasting temperature and a high peak viscosity contribute to the resilience and chewiness of the food.

### 3.2. Texture Properties

The descriptive statistics of textural parameters of Niangao are listed in Table 4 and Table 5. The hardness is the peak force during the first compression cycle of the Niangao using a probe [16]. As the cooked Niangao is sticky in nature, the peak force generally occurs at the point of maximum deformation. The adhesiveness is the negative force at the first bite [16]. The adhesiveness properties provide a highly intuitive reflection of the degree to which the Niangao adheres to the teeth [17].

The CV of cohesiveness was 7.90%, which was the lowest among all these texture properties. The CV of adhesiveness yielded as high as 98.04%, which exhibited a large discreteness. The CV of hardness, gumminess, and chewiness were all above 30%, the data indicated that hardness, adhesiveness, gumminess, and chewiness were significantly different among different cultivars. The discreteness of these texture properties also suggested that 22 cultivars of glutinous rice that were selected for this research were representative samples. Significant differences in the textural properties of the 22 glutinous rice samples were also noted.

### 3.3. PCA of Texture

**Figure 1:**
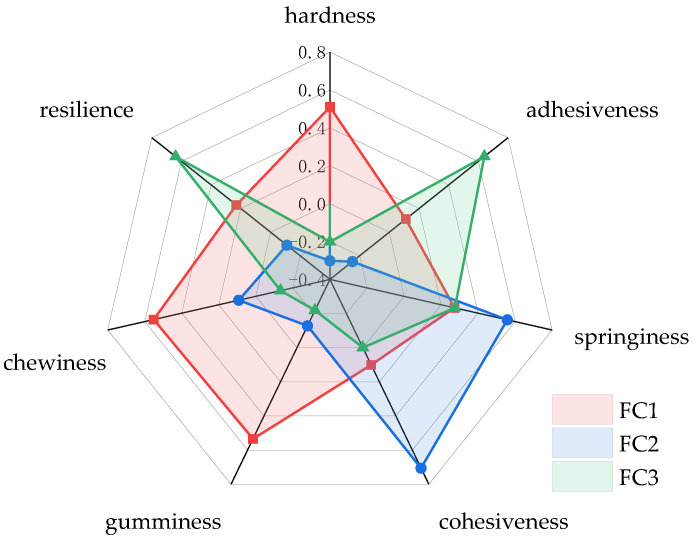
Eigenvectors of PCs.

**Figure 2:**
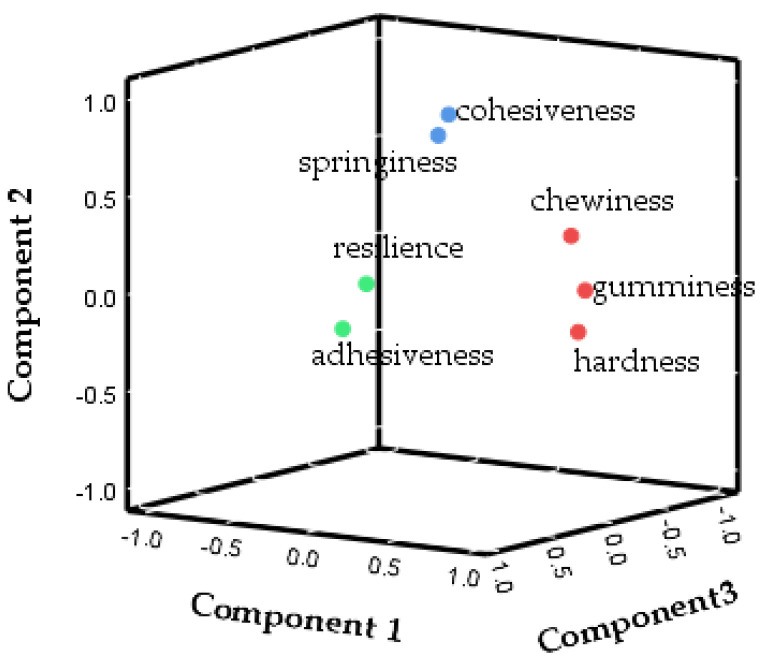
Loadings plot showing the interrelation among the quality attributes of 3 PCs. Colours indicates principal component classification.

The PCs that were extracted for subsequent analysis are presented in Table 6. As can be observed, most of the variation in the data can be explained by the first few principal components. According to the data, the eigenvalues were all above 1.00, three PCs were enough to explain 84.18% of the cumulative variance for Niangao. The cumulative contribution suggested that the first three PCs could represent all the information of textural properties. The coordinate axis in Figure 1 represents the value of eigenvectors of the textural properties. The high value of the eigenvector suggests that the parameter will take more weight in the PC. Hardness, gumminess, and chewiness were represented in PC1; springiness and cohesiveness were represented in PC2; and adhesiveness and resilience were represented in PC3 (Figure 1). Figure 2 shows the 3D loading plot for texture, scores are divided into three different groups. The first one corresponds to adhesiveness and resilience. The second group corresponds to cohesiveness and springiness, and the third one corresponds to hardness, gumminess, and chewiness.

According to the eigenvectors of PCs, linear equations could be constructed.

(1) $$F_{1}=0.510X_{1}+\;0.111X_{2}+\;0.273X_{3}+\;0.101X_{4}+\;0.534X_{5}+\;0.552X_{6}+\;0.230X_{7}$$

(2) $$F_{2}=-0.302X_{1}-\;0.249X_{2}+\;0.558X_{3}+\;0.706X_{4}-\;0.128X_{5}+\;0.093X_{6}-\;0.110X_{7}$$

(3) $$F_{3}=-0.202X_{1}+\;0.640X_{2}+\;0.275X_{3}-\;0.219X_{5}-\;0.133X_{6}+\;0.639X_{7}$$

where F_1_ is the score of PC1, F_2_ is the score of PC2, F_3_ is the score of PC3, X_1_ is the value of hardness, X_2_ is the value of adhesiveness, X_3_ is the value of springiness, X_4_ is the value of cohesiveness, X_5_ is the value of gumminess, X_6_ is the value of chewiness, and X_7_ is the value of resilience. It should be noted that all the values of X are standardized. All three equations were combined as a new equation:

(4) $$F=\frac{\lambda_{1}}{\lambda_{1}+\lambda_{2}+\lambda_{3}}F_{1}+\frac{\lambda_{2}}{\lambda_{1}+\lambda_{2}+\lambda_{3}}F_{2}+\frac{\lambda_{3}}{\lambda_{1}+\lambda_{2}+\lambda_{3}}F_{3}$$

where F is the comprehensive score of all three PCs, *λ*_1_ is the eigenvalue of PC1, *λ*_2_ is the eigenvalue of PC2, and *λ*_3_ is the eigenvalue of PC3. Equation (4) was simplified to be:

(5) $$F=0.151X_{1}+\;0.142X_{2}+\;0.343X_{3}+\;0.227X_{4}+\;0.202X_{5}+\;0.285X_{6}+\;0.238X_{7}$$

Equation (5) is designated as the model in this study. The value of F represents the comprehensive scores of Niangao. A higher value of F suggests that the texture properties are better. The standardized values of textural parameters were substituted into Equation (5), the results are listed in Table 7. The comprehensive score of WKN1 was 1.253, the highest one, which indicated that WKN1 was the best cultivar for producing Niangao. For all samples, comprehensive scores ranged from −2.427 to 1.253. These results revealed that special cultivars of glutinous rice could be found for producing Niangao which has better texture properties.

### 3.4. HCA of Comprehensive Scores

**Figure 3:**
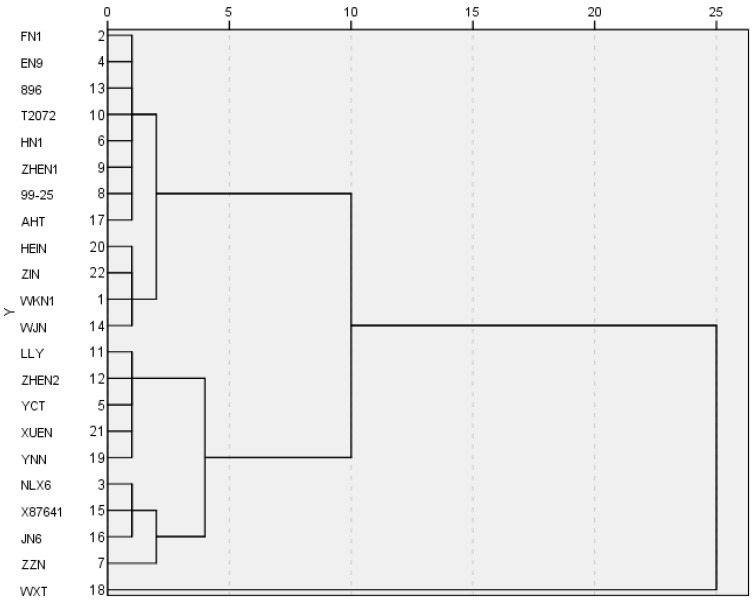
Dendrogram of HCA.

The comprehensive scores of Niangao were classified by using HCA. The dendrogram is shown in Figure 3. Three clusters were obtained based on squared Euclidean distance at five. The first cluster included FN1, EN9, 896, T2072, HN1, ZHEN1, 99-25, AHT, HEIN, ZIN, WKN1, and WJN whose comprehensive scores ranged from 0.281 to 1.253. The second cluster included LLY, ZHEN2, YCT, XUEN, YNN, NLX6, X87641, JN6, and ZZN whose comprehensive scores ranged from −1.689 to 0.044. The third one included WXT whose comprehensive score was −2.427. The results supposed that those cultivars in the second and third clusters were not suitable for producing Niangao. The data indicated that the first cluster could be defined as good materials for Niangao. From the texture properties, it can be observed that the Niangao produced from these varieties of glutinous rice materials exhibits good hardness, adhesiveness, and springiness. In consequence, those 12 cultivars of glutinous rice were extracted for subsequent steps.

### 3.5. SLR Statistic

The comprehensive scores were set as the dependent variable, and standardized indicators were set as the independent variable. The regression model summary is listed in Table 8. The data showed that the model was significant and it could be regarded as one of the criteria for predicting the texture properties of Niangao. The SLR model was constituted by entered variables and constants. The regression equation is as follows:

(6) $$F=0.561{Ζ}_{f\nu }-0.801{Ζ}_{p\nu }+0.747(R^{2}=0.702)$$

where F is the comprehensive score of Niangao, Z_f*ν*_ is the standardized value of the final viscosity of the material, and Z_p*ν*_ is the standardized value of the peak viscosity of the material.

**Figure 4:**
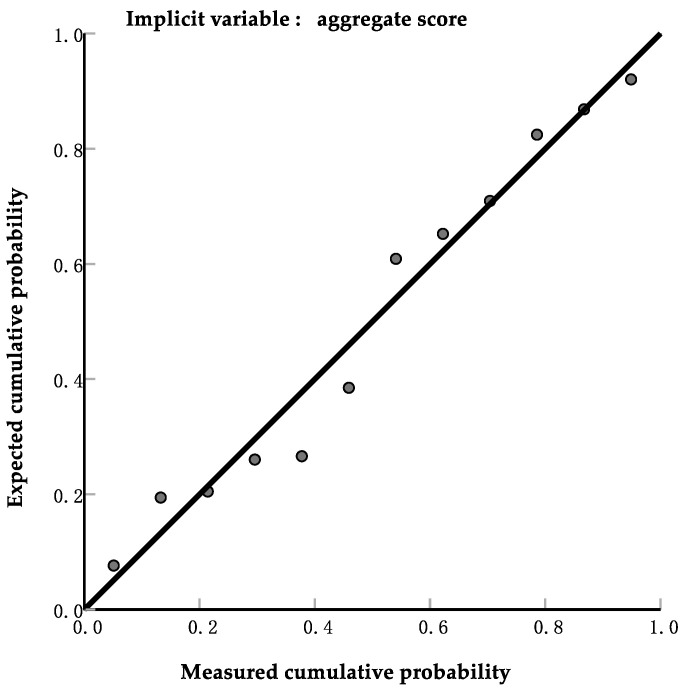
Correlation of comprehensive scores with values calculated using the model equation.

Equation (6) showed that indicators of materials were removed by SLR except peak viscosity and final viscosity. The correlation of the observed comprehensive scores and those predicted by the SLR model Equation (6) based on peak viscosity and final viscosity is shown in Figure 4. The result of SLR demonstrated that the texture properties of Niangao are dependent on peak viscosity and final viscosity, both of which are crucial indicators of glutinous rice for producing Niangao. It was reported that the differences among pasting properties are one of the most sensitive indices in rice grains highly associated with rice cooking and eating quality [18]. The pasting properties of the starch are important indicators of the behavior of the starch during production. Consequently, pasting properties related to molecular structure could be used to decide the suitability of starch for different food products and other related products [19]. The pasting properties are affected by amylose content, amylopectin branch chain length distribution, and molecular size [20].

When starch–water dispersion starts to be heated and subjected to shear forces, starch granules absorb water and swell, resulting in an increase in viscosity. Peak viscosity is indicative of water-binding capacity [21]. A hypothesis was proposed that peak viscosity might mostly be dictated by amylopectin molecular size [22]. In this study, peak viscosity was negatively correlated with the texture quality of Niangao. This is probably due to the small clusters of starch chains or molecules that could be easily dissociated, hydrated, and swollen during gelatinization with excess water, thus contributing to an increase in pasting viscosity [23]. Tester and Morrison reported that swelling of starch granules was inhibited by complexes formed with amylose and lipids which results in a lower peak viscosity, the complexes could significantly affect the texture of products [24]. Final viscosity is the amount of recovery of starch viscosity during cooling of the reheated starch pastes and is thus usually related to the amylose content of starch. It was reported that an increase in amylose content contributes to the formation of a gel network and an increase in gel strength [25]. However, in recent research, since the difference in amylose content of rice starches was marginal, the molecular structure of amylopectin could mainly affect final viscosity. In this study, the final viscosity shows a positive correlation with the texture quality of Niangao. Higher amylopectin molecular weight and long chains could provide strong interaction to maintain the integrity of the swollen granules which would result in some change in the texture of rice products [26]. The chains in amylopectin have a main role in the structure of the crystalline lamellae, which could probably contribute to the reorganization during cooling, nevertheless, the amylopectin short branch chains reduce the efficiency of molecular aggregation during cooling, which could induce a lower final viscosity [27]. The reorganization and aggregation of molecules finally result in the differences in texture properties of Niangao.

## 4. Conclusions

Twenty-two cultivars of glutinous rice were used to produce Niangao. The texture properties of Niangao were analyzed using PCA. The factor structure of PCA revealed three major groups comprising hardness, gumminess, and chewiness on PC1; springiness and cohesiveness on PC2; and adhesiveness and resilience on PC3. In addition, comprehensive scores of Niangao were calculated by mathematical methods to represent the textural properties. A total of 12 cultivars of glutinous rice were extracted by HCA owing to their suitability for producing Niangao. An ensemble model was formed based on SLR. Peak viscosity and final viscosity were significantly correlated to comprehensive scores, and the standard partial regression coefficients were −0.801 and 0.561. The R^2^ of the model was 0.702, and the *p*-value was 0.004. The results showed that this SLR model could well predict the texture quality of Niangao based on the peak viscosity and final viscosity of glutinous rice.

This study provides a method and basis for selecting glutinous rice to produce high-quality flavored rice cakes by identifying the key factors influencing their texture. Different rice varieties have their own advantages and disadvantages as ingredients for Niangao production. On one hand, this implies the necessity of breeding rice varieties specifically for Niangao production. On the other hand, it also suggests that the selection of glutinous rice raw materials and subsequent research for Niangao products should be focused on target markets and consumer preferences.

## Figures and Tables

**Table 1:** Basic chemical composition of glutinous rice raw material.

Glutinous Rice Variety	Short Form Name	Water Content/%	Protein Content/%	Lipid Content/%	Total Starch Content/%	Amylose Content/%
Wankennuo No. 1	WKN1	12.28 ± 0.06 ^m^	8.14 ± 0.17 ^f^	0.94 ± 0.07 ^f^	82.48 ± 1.80 ^d^	1.99 ± 0.10 ^b^
Fengnuo No. 1	FN1	11.52 ± 0.13 ^p^	9.11 ± 0.29 ^a^	0.89 ± 0.09 ^f^	78.84 ± 0.43 ^h^	2.52 ± 0.35 ^b^
Nuoliangxiang No. 6	NLX6	11.77 ± 0.04 ^o^	7.38 ± 0.04 ^i^	1.50 ± 0.13 ^a^	84.86 ± 1.84 ^a^	2.84 ± 0.49 ^a^
Enuo No. 9	EN9	11.75 ± 0.13 ^o^	6.81 ± 0.25 ^l^	0.93 ± 0.09 ^f^	81.09 ± 0.07 ^g^	2.57 ± 0.64 ^a^
YingchentaihuNuo	YCT	14.75 ± 0.06 ^c^	7.47 ± 0.01 ^i^	1.22 ± 0.10 ^a^	83.86 ± 3.15 ^c^	3.71 ± 0.98 ^a^
Hongnuo No. 1	HN1	11.86 ± 0.17 ^o^	8.10 ± 0.01 ^f^	1.40 ± 0.29 ^a^	80.92 ± 2.14 ^g^	2.46 ± 0.11 ^b^
Zhenzhu Nuo	ZZN	13.78 ± 0.01 ^h^	7.63 ± 0.21 ^h^	1.05 ± 0.07 ^c^	81.87 ± 1.89 ^e^	3.01 ± 0.61 ^a^
99-25	99-25	13.35 ± 0.06 ^j^	8.51 ± 0.17 ^e^	0.95 ± 0.12 ^e^	82.00 ± 1.73 ^e^	2.47 ± 0.04 ^b^
Zhènnuo	ZHEN1	13.93 ± 0.04 ^g^	8.64 ± 0.08 ^d^	0.84 ± 0.04 ^g^	84.54 ± 0.60 ^a^	2.73 ± 0.16 ^a^
Tenuo 2072	T2072	12.46 ± 0.02 ^l^	7.85 ± 0.13 ^g^	0.78 ± 0.06 ^h^	85.72 ± 0.63 ^a^	3.10 ± 0.27 ^a^
Liuliangyou	LLY	14.09 ± 0.05 ^e^	7.48 ± 0.08 ^i^	1.04 ± 0.09 ^d^	84.45 ± 0.21 ^b^	2.35 ± 0.13 ^b^
Zhēnnuo	ZHEN2	13.07 ± 0.02 ^k^	7.36 ± 0.04 ^i^	1.03 ± 0.08 ^d^	82.11 ± 1.18 ^e^	2.74 ± 0.12 ^a^
896 Nuo	896	15.79 ± 0.02 ^a^	8.97 ± 0.17 ^b^	0.73 ± 0.27 ^h^	86.48 ± 1.25 ^a^	2.88 ± 0.24 ^a^
Wanjingnuo	WJN	13.54 ± 0.02 ^i^	8.53 ± 0.08 ^e^	1.06 ± 0.06 ^c^	86.09 ± 2.33 ^a^	2.09 ± 1.00 ^b^
Zhongxiannuo 87641	X87641	14.00 ± 0.04 ^f^	7.23 ± 0.04 ^j^	0.79 ± 0.22 ^h^	87.79 ± 2.02 ^a^	2.44 ± 0.80 ^b^
Jingnuo No. 6	JN6	12.08 ± 0.04 ^n^	7.38 ± 0.17 ^i^	0.97 ± 0.09 ^e^	87.76 ± 2.10 ^a^	2.13 ± 0.68 ^b^
Anhuitaihunuo	AHT	14.20 ± 0.04 ^e^	7.25 ± 0.13 ^j^	1.36 ± 0.34 ^a^	87.45 ± 1.39 ^a^	2.50 ± 0.98 ^b^
Wuxuetaihunuo	WXT	13.71 ± 0.01 ^h^	8.00 ± 0.08 ^f^	1.04 ± 0.07 ^d^	85.78 ± 0.51 ^a^	2.54 ± 1.21 ^b^
Yuenannuo	YNN	13.11 ± 0.06 ^k^	7.16 ± 0.04 ^k^	1.28 ± 0.24 ^a^	85.00 ± 1.66 ^a^	2.09 ± 0.66 ^b^
Heinuo	HEIN	15.12 ± 0.06 ^b^	8.73 ± 0.04 ^c^	1.38 ± 0.15 ^a^	82.07 ± 1.43 ^e^	2.93 ± 0.26 ^a^
Xuenuo	XUEN	14.99 ± 0.02 ^b^	8.89 ± 0.01 ^b^	1.49 ± 0.30 ^a^	82.00 ± 0.46 ^e^	0.90 ± 0.16 ^c^
Zinuo	ZIN	14.52 ± 0.02 ^d^	9.29 ± 0.04 ^a^	1.14 ± 0.06 ^b^	81.61 ± 1.69 ^f^	2.96 ± 0.64 ^a^

Data for chemical composition (except moisture) in the table are on a dry basis. The significant differences were represented by lowercase letters including a, b, c, etc., with the same letter indicating no significant difference and different letters indicating significant difference.

**Table 2:** Pasting properties of glutinous rice raw material.

Short Form Name of Variety	Peak Viscosity/cP	Pasting Temperature/°C	Breakdown/cP	Setback/cP	Trough/cP	Final Viscosity/cP
WKN1	1571.33 ± 22.94 ^i^	73.92 ± 0.06 ^h^	445.33 ± 7.23 ^l^	226.00 ± 2.65 ^h^	1126.00 ± 15.87 ^g^	1352.00 ± 13.89 ^g^
FN1	2518.33 ± 19.40 ^c^	82.43 ± 0.42 ^b^	670.67 ± 23.44 ^h^	271.67 ± 5.51 ^f^	1847.67 ± 6.66 ^a^	2119.33 ± 1.15 ^b^
NLX6	2785.33 ± 112.19 ^a^	78.18 ± 0.41 ^d^	1077.67 ± 128.22 ^d^	392.33 ± 59.75 ^b^	1707.67 ± 17.21 ^b^	2100.00 ± 44.24 ^b^
EN9	2503.33 ± 3.51 ^c^	87.57 ± 0.03 ^a^	631.67 ± 16.92 ^h^	426.33 ± 24.95 ^a^	1871.67 ± 15.95 ^a^	2298.00 ± 9.17 ^a^
YCT	2212.33 ± 41.67 ^f^	73.65 ± 0.44 ^h^	850.67 ± 41.36 ^g^	339.67 ± 28.01 ^d^	1361.67 ± 27.47 ^d^	1701.33 ± 20.60 ^d^
HN1	2319.00 ± 24.43 ^e^	78.72 ± 0.73 ^d^	684.33 ± 24.13 ^h^	343.33 ± 24.11 ^d^	1634.67 ± 15.04 ^c^	1978.00 ± 28.79 ^c^
ZZN	2743.33 ± 91.59 ^a^	76.57 ± 0.46 ^f^	1073.33 ± 89.00 ^d^	349.33 ± 48.27 ^d^	1670.00 ± 18.00 ^b^	2019.33 ± 52.39 ^c^
99-25	1584.00 ± 41.58 ^i^	70.97 ± 0.47 ^m^	659.67 ± 30.24 ^h^	197.33 ± 2.52 ^i^	924.33 ± 11.85 ^i^	1121.67 ± 14.22 ^i^
ZHEN1	1194.33 ± 27.30 ^k^	71.48 ± 0.06 ^l^	703.33 ± 21.36 ^h^	138.67 ± 1.15 ^l^	491.00 ± 6.08 ^n^	629.67 ± 6.66 ^m^
T2072	2532.00 ± 7.55 ^b^	80.52 ± 0.88 ^c^	1176.00 ± 2.65 ^c^	365.33 ± 15.63 ^c^	1356.00 ± 6.08 ^d^	1721.33 ± 10.02 ^d^
LLY	2625.67 ± 53.58 ^b^	74.52 ± 0.49 ^g^	1437.67 ± 35.50 ^a^	300.67 ± 11.24 ^e^	1188.00 ± 19.29 ^f^	1488.67 ± 29.74 ^f^
ZHEN2	2405.00 ± 89.07 ^d^	73.38 ± 0.49 ^i^	1434.33 ± 56.52 ^a^	251.33 ± 18.01 ^g^	970.67 ± 32.56 ^h^	1222.00 ± 50.57 ^h^
896	1620.33 ± 42.90 ^h^	74.25 ± 0.91 ^h^	1022.00 ± 28.48 ^e^	209.00 ± 9.64 ^h^	598.33 ± 42.02 ^l^	807.33 ± 50.02 ^k^
WJN	1054.67 ± 56.89 ^l^	71.43 ± 0.20 ^l^	605.00 ± 43.86 ^i^	131.67 ± 6.81 ^m^	449.67 ± 13.65 ^o^	581.33 ± 20.40 ^n^
X87641	1951.33 ± 24.19 ^g^	72.58 ± 0.41 ^j^	978.00 ± 18.52 ^f^	248.33 ± 10.02 ^g^	973.33 ± 5.69 ^h^	1221.67 ± 6.11 ^h^
JN6	2600.00 ± 40.95 ^b^	73.67 ± 0.53 ^h^	1346.00 ± 24.58 ^b^	362.33 ± 17.01 ^c^	1254.00 ± 18.52 ^e^	1616.33 ± 35.13 ^e^
AHT	1311.33 ± 42.67 ^j^	72.62 ± 0.51 ^j^	791.67 ± 21.59 ^g^	143.00 ± 3.46 ^l^	519.67 ± 21.22 ^m^	662.67 ± 23.80 ^l^
WXT	1718.67 ± 25.32 ^h^	73.52 ± 0.49 ^i^	983.33 ± 16.86 ^f^	214.67 ± 7.77 ^h^	735.33 ± 8.62 ^j^	950.00 ± 16.37 ^j^
YNN	2818.00 ± 54.37 ^a^	75.25 ± 0.43 ^g^	1123.67 ± 24.19 ^c^	436.00 ± 12.77 ^a^	1694.33 ± 35.85 ^b^	2130.33 ± 27.06 ^b^
HEIN	1101.67 ± 5.03 ^k^	72.28 ± 0.03 ^k^	555.33 ± 1.15 ^k^	170.67 ± 1.53 ^k^	546.33 ± 5.03 ^m^	717.00 ± 3.61 ^l^
XUEN	959.67 ± 43.66 ^m^	77.65 ± 0.39 ^e^	290.00 ± 15.72 ^m^	176.67 ± 4.93 ^j^	669.67 ± 27.97 ^k^	846.33 ± 32.88 ^k^
ZIN	1197.67 ± 37.63 ^k^	72.30 ± 0.09 ^k^	589.67 ± 20.31 ^j^	180.67 ± 3.06 ^j^	608.00 ± 17.69 ^l^	788.67 ± 20.74 ^k^

The significant differences were represented by lowercase letters including a, b, c, etc., with the same letter indicating no significant difference and different letters indicating significant difference.

**Table 3:** Descriptive statistics of Basic chemical compositions and pasting properties of the raw materials.

	Properties	Minimum	Maximum	Mean	Std. Deviation	CV (%)
Basic chemical compositions	Water content (%)	11.52	15.79	13.44	1.23	9.13
	Protein content (%)	6.81	9.29	8.00	0.73	9.18
	Lipid content (%)	0.73	1.50	1.08	0.23	21.59
	Total starch content (%)	78.84	87.79	83.85	2.50	2.98
	Amylose content (%)	0.90	3.71	2.54	0.54	21.26
pasting properties	Peak viscosity (cP)	959.67	2818.00	1969.42	647.99	32.90
	Pasting temperature (°C)	70.97	87.57	75.34	4.10	5.44
	Breakdown (cP)	290.00	1437.67	869.52	318.59	36.64
	Setback (cP)	131.67	436.00	267.05	96.54	36.15
	Trough (cP)	449.67	1871.67	1099.91	486.32	44.21
	Final viscosity (cP)	581.33	2298.00	1366.95	574.90	42.06

CV = coefficient of variation (%).

**Table 4:** Texture properties of Niangao.

Short Form Name	Hardness/g	Adhesiveness/(g·s)	Springiness	Cohesiveness	Gumminess	Chewiness/g	Resilience
WKN1	6304.63 ± 709.72 ^b^	−6.06 ± 1.07 ^a^	0.92 ± 0.04 ^a^	0.77 ± 0.03 ^c^	4828.71 ± 581.55 ^b^	4430.89 ± 460.71 ^a^	0.37 ± 0.03 ^a^
FN1	4892.52 ± 1079.85 ^f^	−16.52 ± 1.88 ^a^	0.78 ± 0.03 ^b^	0.85 ± 0.03 ^a^	4165.31 ± 899.35 ^d^	3277.15 ± 800.15 ^c^	0.33 ± 0.02 ^d^
NLX6	3710.93 ± 471.63 ^i^	−2.79 ± 0.41 ^a^	0.74 ± 0.04 ^d^	0.68 ± 0.03 ^h^	2517.45 ± 292.53 ^h^	1860.51 ± 241.57 ^g^	0.33 ± 0.03 ^d^
EN9	4706.38 ± 620.28 ^f^	−45.00 ± 3.78 ^c^	0.89 ± 0.06 ^a^	0.74 ± 0.03 ^e^	3467.42 ± 542.06 ^f^	3082.33 ± 411.63 ^d^	0.37 ± 0.04 ^a^
YCT	7063.09 ± 902.58 ^a^	−164.36 ± 20.26 ^g^	0.72 ± 0.06 ^e^	0.73 ± 0.07 ^f^	5156.45 ± 607.42 ^a^	3715.10 ± 611.19 ^b^	0.34 ± 0.01 ^c^
HN1	5786.87 ± 1077.73 ^d^	−213.00 ± 41.01 ^i^	0.92 ± 0.03 ^a^	0.87 ± 0.03 ^a^	5012.78 ± 918.39 ^a^	4575.05 ± 762.03 ^a^	0.30 ± 0.02 ^f^
ZZN	2875.94 ± 272.56 ^k^	−44.94 ± 10.13 ^c^	0.67 ± 0.04 ^g^	0.81 ± 0.06 ^a^	2322.83 ± 153.69 ^h^	1556.23 ± 115.22 ^i^	0.25 ± 0.02 ^j^
99-25	7779.47 ± 554.76 ^a^	−81.83 ± 6.63 ^e^	0.86 ± 0.04 ^a^	0.66 ± 0.03 ^i^	5153.26 ± 343.37 ^a^	4448.15 ± 500.49 ^a^	0.37 ± 0.01 ^a^
ZHEN1	8573.73 ± 1153.52 ^a^	−36.08 ± 6.11 ^a^	0.68 ± 0.09 ^f^	0.74 ± 0.03 ^e^	6395.38 ± 1006.16 ^a^	4307.02 ± 862.83 ^a^	0.41 ± 0.02 ^a^
T2072	4271.39 ± 645.24 ^g^	−82.69 ± 10.73 ^f^	0.89 ± 0.06 ^a^	0.84 ± 0.04 ^a^	3602.50 ± 602.43 ^e^	3227.05 ± 647.42 ^c^	0.33 ± 0.02 ^d^
LLY	4436.56 ± 1743.67 ^g^	−141.21 ± 30.60 ^g^	0.75 ± 0.04 ^c^	0.78 ± 0.03 ^b^	3491.73 ± 1421.55 ^e^	2658.44 ± 1112.39 ^f^	0.34 ± 0.01 ^c^
ZHEN2	5809.33 ± 322.51 ^c^	−15.58 ± 6.10 ^a^	0.67 ± 0.05 ^g^	0.74 ± 0.03 ^e^	4318.17 ± 300.36 ^c^	2870.65 ± 152.31 ^e^	0.33 ± 0.03 ^d^
896	3745.72 ± 620.61 ^i^	−56.17 ± 10.95 ^d^	0.85 ± 0.03 ^a^	0.85 ± 0.03 ^a^	3212.20 ± 620.33 ^g^	2735.92 ± 514.59 ^e^	0.37 ± 0.02 ^a^
WJN	5168.44 ± 1539.55 ^e^	−132.72 ± 51.69 ^g^	0.90 ± 0.07 ^a^	0.87 ± 0.05 ^a^	4533.91 ± 1608.95 ^c^	4123.03 ± 1709.65 ^a^	0.38 ± 0.03 ^a^
X87641	4083.18 ± 325.91 ^h^	−251.27 ± 28.51 ^j^	0.75 ± 0.02 ^c^	0.78 ± 0.05 ^b^	3176.38 ± 353.43 ^g^	2364.85 ± 219.92 ^f^	0.29 ± 0.01 ^g^
JN6	3334.34 ± 632.04 ^j^	−17.76 ± 3.63 ^a^	0.75 ± 0.06 ^c^	0.73 ± 0.03 ^f^	2412.99 ± 410.48 ^h^	1809.31 ± 310.33 ^h^	0.36 ± 0.02 ^b^
AHT	3919.51 ± 902.09 ^i^	−1.54 ± 0.36 ^a^	0.84 ± 0.06 ^a^	0.85 ± 0.01 ^a^	3352.68 ± 826.95 ^f^	2790.59 ± 474.06 ^e^	0.40 ± 0.02 ^a^
WXT	2877.25 ± 431.78 ^k^	−181.33 ± 32.87 ^h^	0.65 ± 0.05 ^h^	0.70 ± 0.02 ^g^	2002.27 ± 329.36 ^i^	1292.08 ± 157.26 ^j^	0.28 ± 0.03 ^h^
YNN	5993.78 ± 593.95 ^c^	−34.54 ± 3.02 ^a^	0.77 ± 0.03 ^b^	0.76 ± 0.04 ^d^	4536.61 ± 629.42 ^c^	3505.02 ± 500.67 ^b^	0.32 ± 0.02 ^e^
HEIN	7387.79 ± 764.52 ^a^	−7.02 ± 0.64 ^a^	0.82 ± 0.03 ^a^	0.78 ± 0.04 ^b^	5739.40 ± 716.34 ^a^	4677.61 ± 575.96 ^a^	0.37 ± 0.01 ^a^
XUEN	6849.53 ± 367.35 ^a^	−76.94 ± 9.20 ^e^	0.66 ± 0.05 ^h^	0.79 ± 0.02 ^b^	5383.90 ± 323.97 ^a^	3575.01 ± 373.54 ^b^	0.30 ± 0.02 ^f^
ZIN	7563.27 ± 1153.95 ^a^	−42.61 ± 3.35 ^b^	0.87 ± 0.05 ^a^	0.82 ± 0.01 ^a^	6209.52 ± 926.75 ^a^	5415.37 ± 1065.81 ^a^	0.26 ± 0.02 ^i^

The significant differences were represented by lowercase letters including a, b, c, etc., with the same letter indicating no significant difference and different letters indicating significant difference.

**Table 5:** Descriptive statistics of textural parameters of Niangao.

Parameters	Minimum	Maximum	Mean	Std. Deviation	CV (%)
hardness (g)	2875.94	8573.73	5324.26	1691.80	31.78
adhesiveness (g·s)	−251.27	−1.54	−75.09	73.62	98.04
springiness	0.65	0.92	0.79	0.09	11.57
cohesiveness	0.66	0.87	0.78	0.06	7.90
gumminess	2002.27	6395.38	4135.99	1275.66	30.84
chewiness (g)	1292.09	5415.37	3286.24	1113.57	33.89
resilience	0.25	0.41	0.34	0.04	12.85

CV = coefficient of variation (%).

**Table 6:** Eigenvalues and variance contributions of PCs.

PC	Extraction Sums of Squared Loadings		
	Eigenvalue	Contribution of Variance (%)	Cumulative Contribution (%)
1	3.12	44.59	44.59
2	1.45	20.66	65.25
3	1.33	18.93	84.18

Extraction Method: PCA.

**Table 7:** Comprehensive scores (F) of Niangao.

Materials	Comprehensive Score	Materials	Comprehensive Score
WKN1	1.253	ZHEN2	−0.537
FN1	0.285	896	0.305
NLX6	−1.200	WJN	1.124
EN9	0.281	X87641	−1.276
YCT	−0.154	JN6	−0.901
HN1	0.835	AHT	0.587
ZZN	−1.689	WXT	−2.427
99-25	0.676	YNN	0.044
ZHEN1	0.847	HEIN	1.206
T2072	0.388	XUEN	−0.242
LLY	−0.580	ZIN	1.176

**Table 8:** Model summary.

Method	R	R^2^	Sig.
Stepwise (Criteria: Probability-of-F-to-enter ≤ 0.050, Probability-of-F-to-remove ≥ 0.100)	0.838	0.702	0.004 **

Criteria: Use probability of F; **: Significant at *p* < 0.01.

## Data Availability

The original contributions presented in the study are included in the article, further inquiries can be directed to the corresponding author.
